# COVID-19 in conflict border regions: a case of South Kordofan, Sudan

**DOI:** 10.1186/s13031-021-00370-9

**Published:** 2021-05-04

**Authors:** Quraish Sserwanja, Mohammed Bashir Adam, Joseph Kawuki, Emmanuel Olal

**Affiliations:** 1 Programmes Department, GOAL Global, Khartoum, Sudan; 2 Global Health Uganda, Mawanda Road, Plot 667, P.0 Box 33842, Kampala, Uganda; 3 Centre for Health Behaviors Research, Jockey Club School of Public Health and Primary Care, The Chinese University of Hong Kong, Hong Kong- SAR, China; 4 Yotkom Medical Centre, Kitgum, Uganda

**Keywords:** COVID-19, Post‐conflict, Rural, Sudan and South Kordofan

## Abstract

The novel coronavirus disease (COVID-19) was first reported in Sudan on 13 March 2020. Since then, Sudan has experienced one of the highest rates of COVID-19 spread and fatalities in Africa. One year later, as per 22 March 2021, Sudan had registered 29,661 confirmed cases and 2,028 deaths with a case fatality rate (CFR) of 6.8 %. By 12 December 2020, of the 18 states in Sudan, South Kordofan had the fifth highest CFR of 17.4 %, only surpassed by the other conflict affected North (57.5 %), Central (50.0 %) and East (31.8 %) Darfur States. By late March 2021, just three months from December 2020, the number of cases in South Kordofan increased by 100 %, but with a significant decline in the CFR from 17.4 to 8.5 %. South Kordofan is home to over 200,000 poor and displaced people from years of destructive civil unrests. To date, several localities such as the Nubba mountains region remain under rebel control and are not accessible. South Kordofan State Ministry of Health in collaboration with the federal government and non-governmental organizations set up four isolation centres with 40 total bed capacity, but with only two mechanical ventilators and no testing centre. There is still need for further multi-sectoral coalition and equitable allocation of resources to strengthen the health systems of rural and conflict affected regions. This article aims at providing insight into the current state of COVID-19 in South Kordofan amidst the second wave to address the dearth of COVID-19 information in rural and conflict affected regions.

## Introduction

Globally, several socioeconomic and livelihood activities have been disrupted by lockdown measures to control Corona virus disease 2019 (COVID-19) spread since it was declared a global pandemic by the World Health Organization on 11 March 2020 [1, 2]. These disruptions have further strained the already weak health systems of many low- and middle-income economies [1–3]. In Africa, Sudan was among the first countries to report COVID-19 cases in early March 2020 [4]. Sudan is the second largest country in Africa by land mass, with a population of over 40 million people [4, 5]. It is a refugee hosting country for thousands of refugees from South Sudan, Somali, Eritrea, Central African Republic, Syria, Chad, Yemen, and Ethiopia [4–6]. Despite high COVID-19 case fatality rate(CFR), Sudan has had limited national capacity to effectively manage patients in need of advanced respiratory support and critical care. South Kordofan is located in the South Central region of Sudan, with an estimated population of 2.5 million people [7]. It is a wide conflict affected State bordering South Sudan in the South. The years of conflict in the region has resulted in displacement of over 200 000 people, extensive infrastructure destruction and high poverty levels [7, 8].

Besides COVID-19, South Kordofan has previously been affected by yellow fever, chikungunya and dengue fever epidemics, which immensely strained the health system [9, 10]. An inter-agency assessment report in May 2020 in the internally displaced people’s camps found overcrowding, poor water supply, inadequate medicines and limited health workers in the State which are risk factors for COVID-19 spread [11].

The second COVID-19 wave is anticipated to further strain health services in the State. Additionally, there might be equal increase in demand for health care by returnees in the event of a successful conclusion of the ongoing peace talks between the new government and the rebels in the region [12], thus making it imperative for the State to effectively plan for the limited resources to ensure proper service delivery in terms of water, sanitation, and hygiene (WASH), health services, and nutrition.

Rural areas in Africa such as South Kordofan, which make up the largest part the continent have been given less attention in the fight against COVID-19 compared to urban areas [13]. With such little attention, these areas continue to face challenges such as; poor road network, insecurity, inadequate health workers, health facilities and inadequate COVID-19 diagnostic services [13, 14].

Of the 18 Sudan States, South Kordofan is among the most under resourced which greatly limits access and provision of essential services [15]. It is therefore crucial to give more attention to these areas to ensure equitable access to care, universal health coverage and above all, enable successful containment of COVID-19.

### Current situation and COVID-19 statistics

The limited testing and screening services in Sudan risks a high number of undetected cases. A recent report showed that between April and September 2020, about 16,090 deaths were undetected in Khartoum and the fatality rates are expected to increase in the second wave compared to the first wave [16]. As per 22 March 2021, Sudan had registered 29,661 confirmed cases and 2,028 deaths with a case fatality rate (CFR) of 6.8 % [17, 18]. Although Khartoum State, the capital, accounts for majority of the cases, most of the COVID-19-related deaths have been reported from other states [17]. Between November and December 2020, Sudan registered a surge in COVID-19 cases, with a rapid increase from 10 cases per day at the beginning of November to about 200–300 cases a day in December [17]. However, since end of December 2020, daily COVID-19 numbers begun to steadily decline to a daily average of about 25 cases from the beginning of February 2021 [17].

The overall CFR as per 22 March 2021 in South Kordofan was about double that of Khartoum, 8.5 % as compared to 4.1 % [18, 19]. Most COVID-19 cases (78.7 %) in South Kordofan have been from communities, and despite the overall decline in CFR from 17.4 % in December 2020 to 8.5 % in March 2021, the state registered 100 % increase in the number of cases within three months from December 2020 to March 2021 [20]. Neighboring conflict prone states have registered similar transmission rates with persistent high CFRs. Currently, the CFRs in North Darfur, Central Darfur and East Darfur States are 45.9 %, 42.9 and 23.5 % respectively ranking as top three in Sudan. These States are as well affected by recurrent conflicts both internally and from bordering Central African Republic and Chad.

### COVID-19 prevention measures

The State Ministry of Health (SMoH) through support from the Federal Ministry of Health (FMoH) and health non-governmental organizations (NGOs) has taken some measures to ensure that COVID-19 is controlled. Due to the scarcity of hospitals in South Kordofan most patients’ first entry points are Primary Health Care facilities which are mainly supported by international non-governmental organizations (INGOs). INGOs mainly provide training, supplies for triage, and COVID-19 related management protocols, referral services, personal protective equipment (PPEs), and infection prevention and control (IPC).

Some of the measures that have thus far been implemented include training and setting up of atleast14 rapid response teams (RRTs). The RRTs are engaged in contact tracing and community-based surveillance. Similarly, there has been continued community awareness and sensitization on COVID-19 preventive measures through mass media campaigns using vehicles with mounted microphones. To supplement nationwide media messages about COVID-19, broadcasting awareness messages via television and local radio stations is ongoing albeit not accessible to everyone. INGOs have also supported the SMoH in implementing community health activities such as community lead action training for local leaders to spearhead COVID-19 community prevention [21]. This approach has been documented to empower communities into taking action against COVID-19 spread [21].

However, the nationwide restriction of movement between States that was imposed from May 2020 to September 2020 was poorly adhered to due to the limited resources to monitor and effect it, and negative attitude of the population towards COVID-19 existence [5]. Nevertheless, there is no clear information regarding the COVID-19 measures in the closed rebel-controlled areas.

Other challenges faced include social distancing being poorly adhered to in the community due to social norms, which are being challenged through community awareness and sensitization messages.

In addition, use of face masks is poorly being adhered to by the community partly due to the negative attitude and limited availability and affordability of face masks. INGOs have been pivotal in supplying PPEs to health facilities to protect essential workers.

### COVID-19 treatment facilities

Although the government of Sudan has set up 36 isolation centres across all the States with a bed capacity of 985 beds and 198 intensive care unit beds, these are still very low as per international standards [5]. South Kordofan SMoH has four isolation centres with a total of 40 bed capacity of which two are intensive care beds with only two mechanical ventilators [22]. Through the support of FMoH and INGOs, health facilities have been trained and supplied with PPEs. There are presently no COVID-19 testing centres in South Kordofan , instead any collected sample is transported through over 500 km to the national capital, Khartoum. This causes delay in receiving results. No clear information is available regarding the COVID-19 treatment services set up in the closed rebel-controlled areas.

The above undertaken preventive measures, to a large extent, have helped to slow down the mortality rate of the disease as indicated by the declining trends in mortality statistics. However, the same is not observed with transmission as evidenced by the significant increase in the number of cases during the second wave. Nevertheless, these statistics could be challenged due to limited testing, diagnosis, and surveillance capacity of the fragile South Kordofan State.

In May 2020, the Inter Agency Standing Committee issued interim guidelines on key public health and social measures needed to reduce the risk of COVID-19 spread and its impact in low capacity and humanitarian settings [23]. Based on these guidelines, South Kordofan has registered some success in particular fields, while still facing challenges in several domains. Commendable efforts have been attained in mobilizing stakeholders and community efforts. In addition, training of health care workers on COVID-19 standard operating procedures and supplying PPEs to health workers has been undertaken by SMoH with support from partners [24, 25]. However, several limitations still exist in complying to social and physical distancing, securing handwashing facilities in public places and inadequate PPE, especially masks in the community. Moreover, screening and testing capacity is also limited due to less funding and resources. Having designated isolation and quarantine facilities in all localities is also a challenge, in addition to the few intensive care units needed for treatment of ill COVID-19 patients. These still are attributed to under financing and lack of structural facilities due to political instability.

### Public Health implications

To ensure effective and well-coordinated response to epidemics, decision-makers need to have the right information via health information systems and data platforms [26]. In South Kordofan and similar contexts, data collection and surveillance are affected by poor internet access, limited phone network coverage and poor roads. This leads to insufficient reporting and delayed response to active cases, risking further spread and mortality. Investing in infrastructure development such as roads and telecommunication will enhance earlier detection and effective response to future epidemics. This will ensure innovations in the health system hence enabling digital data collection, confidential self-reporting, and epidemic control measures such as health messaging [26]. Furthermore, with timely reporting of accurate data, the different stakeholders can ensure efficient resource allocation due to good visibility across the health system’s various components via quality health information systems. However, to ensure that these developments are feasible and sustainable, there is need to promote truce between warring factions during pandemic times and unconditional access to affected populations. Such ceasefire allows vital services and control measures to be availed to all populations through trusted channels.

Conflict affected areas are usually lacking in sectors such as education, gender equality and technology which is the case in South Kordofan compared to other States in Sudan. To ensure effective and context specific health policies, scientifically based evidence is critical. Decision makers and supporting partners should strive to foster a peaceful environment to attract investment in research and economic development. Given the conservative nature of Sudan, research and development strategies need to address gender differences. This is crucial in ensuring feasibility of data collection and ensuring accurate data as women would not readily participate in male dominated activities that would require them being open and giving information.

In conflict-affected and rural communities like South Kordofan mistrust is often high. Community engagement through use of local health committees, trusted leaders, and community health workers is crucial in health service delivery [26, 27]. These help to form a quick link between clinical and community-based services, disseminate culturally appropriate and context specific information on behavior change, which ensures positive responses and community ownership of service delivery. Increased community participation in health projects facilitates the community to contribute to the logistics of the effort and ensures sustainability of the projects or response activities. Stakeholders need to ensure that more trusted members of the community are trained to ensure availability of enough community health workers. Furthermore, their services can be integrated to include nutrition, maternal and child health services. To ensure availability of resources and tackle shortage of staff and supplies, there is still need for multi-sectoral participation in the fight against COVID-19.

Likewise, national pandemic task forces need to have representation from the different regions of the country and establish regular interaction with the different States’ task forces. This will ensure equitable resource allocation and context-specific measures. Multi-sectoral and proper coordination with different stakeholders also ensures pooling of resources especially in such areas that are largely under resourced. Furthermore, decision makers should ensure equitable allocation of resources to rural and conflict-affected areas given their vulnerability. Presently, much of the resources for COVID-19 control are concentrated in Khartoum the capital State, despite much higher CFR in rural and conflict -affected areas such as Darfur and Kordofan.

The dramatic increase in the number of cases observed with the second wave clearly shows a high need to revise the implemented COVID-19 prevention measures in the state and to have regular monitoring and evaluation of the public's knowledge, attitude, and practice regarding the precautionary measures. Further analysis of the registered cases in the second wave could possibly explain the very high transmission rate and low mortality rate and hence facilitating better planning for the next waves.

## Conclusions

This article has provided insight in health system gaps in abating the second wave of COVID-19 in a conflict -affected region of Sudan. In this regard, the article has noted a high case fatality rate in the first wave which significantly declined by a half in the second wave. Generally, although South Kordofan registered a significant decline in the CFR, similar conflicted-affected border regions in Darfur maintained the highest CFRs and yet these regions have limited resources to handle COVID-19 cases in terms of testing and treatment. There is still a need for multi-sectoral participation in the fight against COVID-19 with stronger emphasis put on risk assessments and understanding region-specific COVID-19 dynamics through more research. The FMoH should provide more resources to ensure setting up of regional testing and isolation centres, as well as intensive care unit beds. There is further need to plan effectively for the internally-displaced people's camps and returnees who are expected to further strain the already struggling health system.

## Data Availability

Not applicable.

## Abbreviations

WHO: World Health Organization
ARI: Acute respiratory infections
COVID-19: Coronavirus disease 2019
IPC: Infection prevention and control
PPE: Personal Protective Equipment
ICU: Intensive Care Unit
FMoH: Federal Ministry of Health
SMoH: State Ministry of Health
RRTs: Rapid Response Teams
NGO: Non-Governmental Organizations
